# Advances in Surface Enhanced Raman Spectroscopy for *in Vivo* Imaging in Oncology

**DOI:** 10.7150/ntno.62970

**Published:** 2022-01-01

**Authors:** Kenry, Fay Nicolson, Louise Clark, Sajanlal R. Panikkanvalappil, Bohdan Andreiuk, Chrysafis Andreou

**Affiliations:** 1 Department of Imaging, Dana-Farber Cancer Institute and Harvard Medical School, Boston, MA 02215, USA.; 2 Harvard John A. Paulson School of Engineering and Applied Sciences, Harvard University, Cambridge, MA 02138, USA.; 3 Department of Cancer Biology, Dana-Farber Cancer Institute and Harvard Medical School, Boston, MA 02215, USA.; 4 Department of Cancer Immunology and Virology, Dana-Farber Cancer Institute and Harvard Medical School, Boston, MA 02215, USA.; 5 Department of Electrical and Computer Engineering, University of Cyprus, Nicosia, Cyprus.

## Abstract

In the last two decades, the application of surface enhanced Raman scattering (SERS) nanoparticles for preclinical cancer imaging has attracted increasing attention. Raman imaging with SERS nanoparticles offers unparalleled sensitivity, providing a platform for molecular targeting, and granting multiplexed and multimodal imaging capabilities. Recent progress has been facilitated not only by the optimization of the SERS contrast agents themselves, but also by the developments in Raman imaging approaches and instrumentation. In this article, we review the principles of Raman scattering and SERS, present advances in Raman instrumentation specific to cancer imaging, and discuss the biological means of ensuring selective *in vivo* uptake of SERS contrast agents for targeted, multiplexed, and multimodal imaging applications. We offer our perspective on areas that must be addressed in order to facilitate the clinical translation of SERS contrast agents for *in vivo* imaging in oncology.

## Introduction

The clinical pathway of cancer management relies heavily on the use of medical imaging. Imaging is essential in all aspects of the process, including for initial diagnosis of the primary tumor, treatment planning, and monitoring, often post-therapy, to determine the progression, recurrence, or subsequent growth of metastatic lesions. Continued development and progress within the field of imaging is vital in improving the modern medical care of patients - especially in oncology, where the ability to highlight all nodes of disease, early, efficiently, and precisely, provides a clear advantage in prognosis.^1^ In this review, we will describe the imaging approach and recent advances in preclinical imaging using surface enhanced Raman scattering (SERS) nanoparticles for *in vivo* cancer imaging applications, and discuss how this method can be translated to the clinic.

Initial exploratory imaging, often performed with x-ray or ultrasound, probes only the gross anatomy and reveals the presence of abnormalities. To assess whether a growth is malignant, these imaging modalities are used to guide tissue biopsy for histological analysis - the gold standard of cancer diagnostics. However, imaging alone can provide valuable information when it is performed with enhanced contrast and molecular targeting. The case of ultrasound imaging provides a useful paradigm: innate tissue contrast is weak, and localization of cancerous lesions is vastly improved with the introduction of contrast agents. Microbubbles injected intravenously enable the characterization of focal liver lesions under ultrasonic interrogation. Real-time assessment can be obtained, as benign lesions maintain microbubble enhancement, whilst malignancies show a lack of enhancement, which continues through to the late, hepatic sinusoids phase. This technique of contrast enhanced ultrasound provides a ~70% increase in the confidence of a definite diagnosis, especially for the detection of early stage lesions ≤1 cm in size.^2^ Furthermore, microbubble surface tailoring with targeting ligands has led to molecularly targeted ultrasonography, with the first in-human clinical trials aiming to detect neoangiogenesis using vascular endothelial growth factor receptor type 2 (VEGFR2) labelled microbubbles.^3^

Exogenous contrast agents are inherent in the use of another non-invasive, real-time technique: positron emission tomography (PET) imaging. PET utilizes radioactive tracers for functional imaging, a valuable tool for cancer staging prior to, and during, treatment. The commonly selected radiotracer 2-[^18^F]fluoro-2-deoxy-D-glucose ([^18^F]FDG) highlights areas of a high metabolic turnover rate, often synonymous with carcinomas and metabolic abnormalities indicating pre-malignancy; however, sites of inflammation also demonstrate high metabolism, leading to false positives. Molecularly targeted radiotracers are employed in immuno-PET, and can provide a highly specific localization of cancer related markers.^4^ As biological tissues provide no intrinsic contrast for PET, it provides no physiological information; but when combined with structural imaging method like computed tomography (CT), the multimodal imaging technique PET/CT is able to distinguish metastatic lesions above the millimeter range from the surrounding benign anatomy during the same procedure.^5^ Yet to be brought into the clinic, PET/magnetic resonance imaging (MRI) offers an even greater potential for determining the localization of lesions with higher resolution.^6^

This shift towards using contrast agents for molecular targeting and multimodal techniques has been seen across the entire pre-clinical imaging landscape, in particular when utilizing non-destructive optical imaging methods. Compared to other imaging modalities, optical imaging provides considerably higher spatial resolution and requires smaller and more cost-effective equipment. However, as light does not readily travel through tissue, optical imaging *in vivo* is performed via endoscopes, or in an intraoperative setting. Additionally, near-infrared (NIR) fluorescent agents are preferred, as they allow deeper tissue penetration and lower background autofluorescence compared to visible wavelengths. For example, peptides labeled with fluorescein isothiocyanate (FITC) have been shown to improve diagnosis confidence of Barret's esophagus using a multimodal optical endoscope.^7^ NIR imaging with indocyanine green (ICG) phase enhancement, in combination with mammography and gadolinium-enhanced MRI, has been explored for breast cancer lesion discrimination.^8^, ^9^ Despite enabling molecular targeting, NIR fluorescent agents have wide, overlapping emission bands, which limit their ability to provide multiplexed signals detailing the landscape of heterogenous tumor tissue. Moreover, typical fluorescent agents only provide optical signal, and do not readily translate across multiple modalities. More complex structures, such as nanoparticles, can encapsulate contrast agents for optical imaging and other modalities, providing a platform to enable molecular targeting, and ultimately expand the arsenal of medical imaging capabilities against cancer.

One highly promising nanoparticle-based optical imaging modality is SERS. SERS employs plasmonic nanoparticles and the Raman effect to provide a highly intense and recognizable fingerprint-like spectrum. This technique has shown clinical promise in its ability to delineate microscopic tumors and determine dysplastic precursor lesions and malignant nodes of disease. SERS nanoparticles provide a platform with many opportunities: unsurpassed sensitivity by using molecular resonance effects in surface enhanced resonance Raman spectroscopy (SERRS); active molecular targeting via surface functionalization with targeting moieties; multiplexed imaging, as the distinct spectra emanating from Raman reporter molecules serve to reveal a specific marker; and multimodality as the metal (typically gold) core may serve simultaneously as a CT contrast agent, while PET radioisotopes can be chelated on the surface.

**Figure 1:**
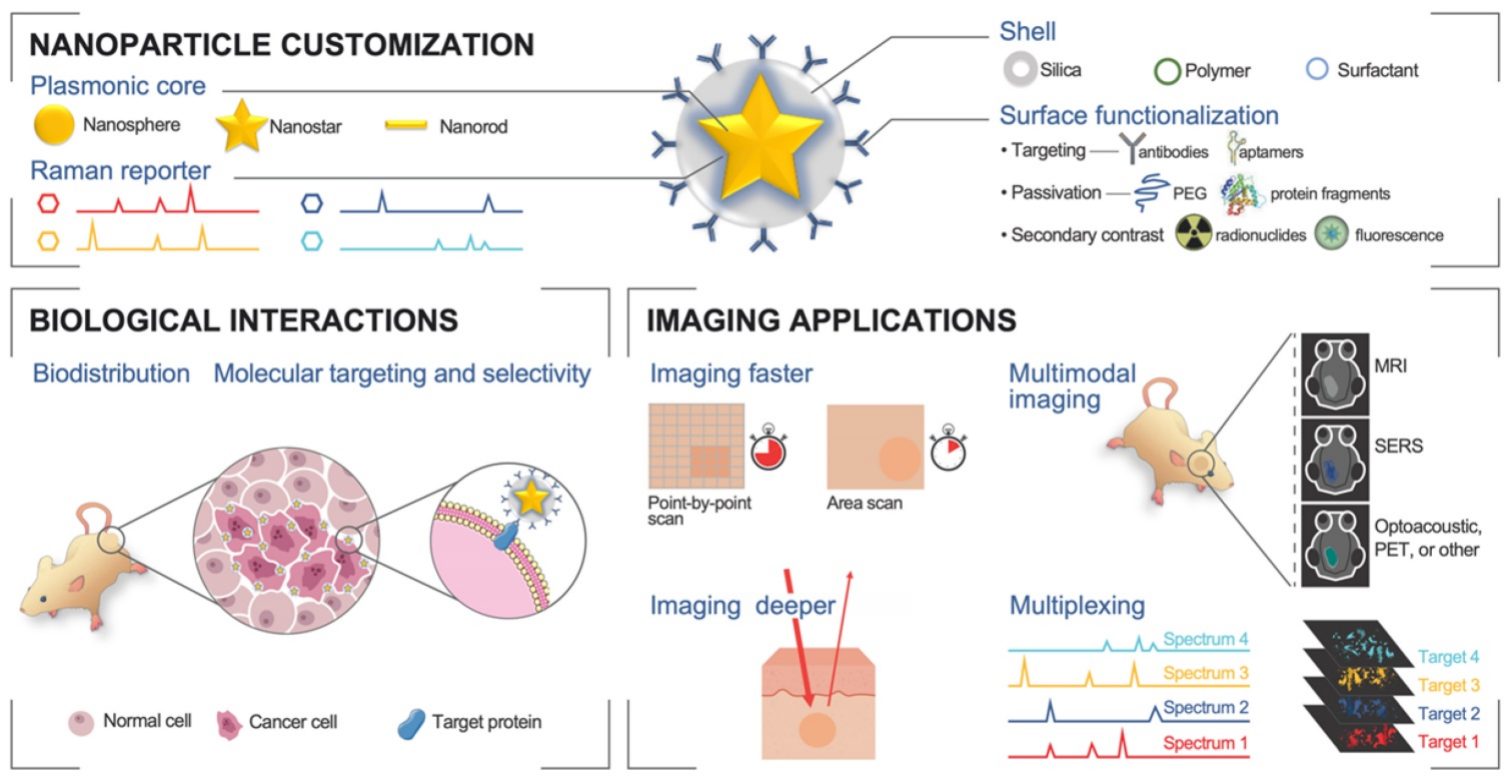
** Advances in SERS-based *in vivo* imaging.** Top: The modular core-shell structure allows nanoparticle customization for strong SERS signal and functionalization for molecular targeting and multimodal contrast. Bottom left: Biological considerations improve nanoparticle pharmacodynamics and allow tumor selectivity and molecular targeting. Bottom right: New instrumentation and processes can lead to improved Raman imaging, as well as multimodal and multiplexed *in vivo* imaging.

In this review, we present recent developments of the methodology of cancer imaging with SERS nanoparticles, focusing on *in vivo* applications. This topic was pioneered by the groups of Dr. Sam Gambhir and Dr. Moritz Kircher, to whose memory this manuscript is dedicated. We will provide a brief theoretical background related to the development of the nanoparticles, examine advances in instrumentation necessary for clinical translation of the method, explore passive and active tumor targeting, and finally consider multimodal and multiplexed imaging strategies. A schematic illustration of the topics covered in our review is shown in **Fig. 1**.

## Principles of Raman Scattering, Surface Enhanced Raman Scattering (SERS), and Surface Enhanced Resonance Raman Scattering (SERRS)

**Figure 2:**
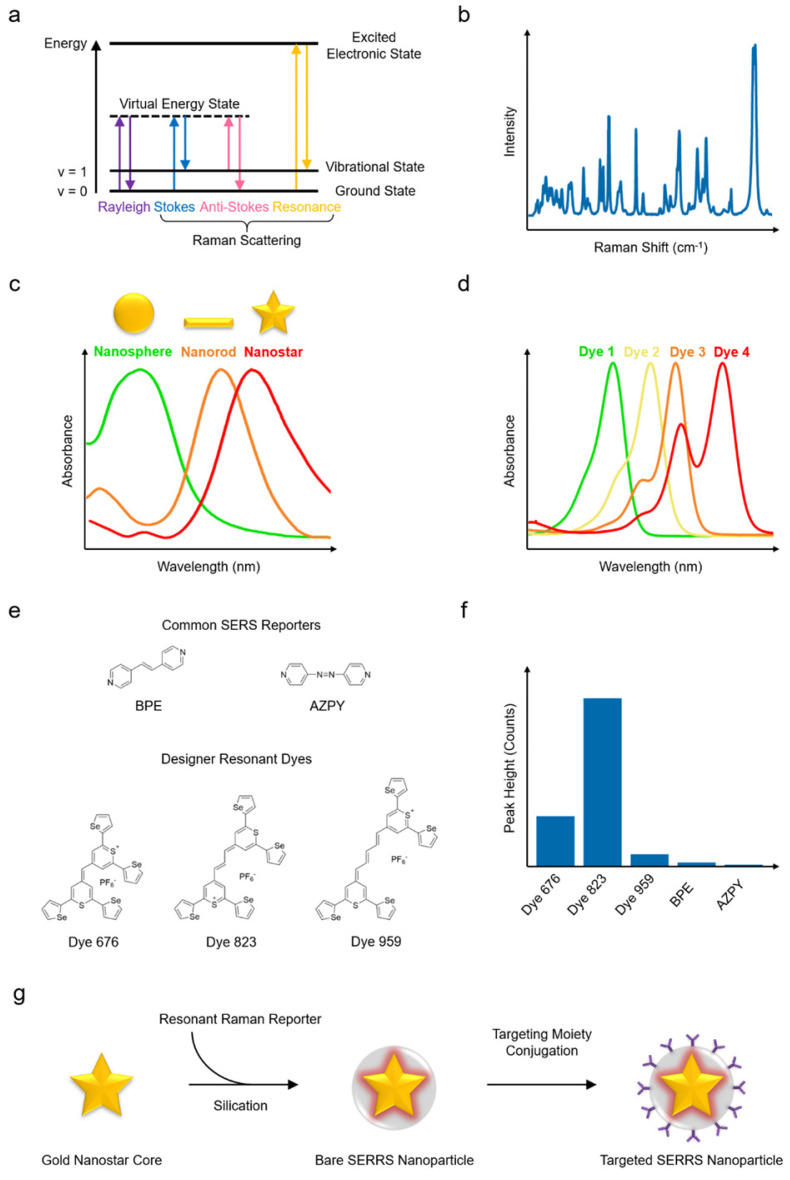
** Principles of Raman scattering, SERS, SERRS, and synthesis of a SERRS nanoparticle.** (a) Jablonski diagram illustrating Raman scattering. (b) The Raman spectrum (“fingerprint”) of a compound has peaks corresponding to the chemical structure. (c) Gold nanostructures with their typical absorption spectra. (d) Examples of fluorescent dye absorption spectra. (e) Chemical structures of example chalcogenopyrylium-based Raman reporters (Dye 676, Dye 823, Dye 959 with optical absorbance at 676, 823, and 959 nm, respectively) and non-resonant reporters (BPE and AZPY). (f) Raman peak intensities of the reporters in (e) excited with an 830 nm laser source. (g) Schematic illustrating the different components of a SERRS nanoparticle and its synthesis process. Both the gold nanostar and the Raman reporter feature absorption maxima in the NIR. (e-f) Adapted with permission from ref. 58. Copyright 2018 The Royal Society of Chemistry.

When light interacts with matter, photons can be scattered elastically or inelastically. Most photons are scattered elastically (i.e., with no energy exchange) in what is termed Rayleigh scattering. However, a small population of photons (about 1 in 10^7^ photons) are scattered inelastically, that is, the photons exchange energy with the scattering material via vibrational transitions within the molecule - this phenomenon is known as the Raman effect, or simply Raman scattering. The Raman scattered photons can lose or gain energy (i.e., they undergo Stokes or anti-Stokes Raman scattering, respectively) as shown in **Fig. 2a**.^10^-^12^ Stokes Raman scattering takes place when the frequency of the scattered photon is lower than that of the incident photon, resulting in the molecule being shifted to a higher vibrational state (e.g., from v = 0 to 1). Conversely, if the frequency of the scattered photon is higher than that of the incident photon and the molecule shifts to a lower energy state (e.g., v = 1 to 0), anti-Stokes Raman scattering occurs. Since the vibrational transitions and related energy exchanges are specific to the molecular composition of the scattering material, the resulting Raman spectrum can be used as a fingerprint to infer detailed structural and chemical information related to the scattering material (**Fig. 2b**). Raman scattering, nevertheless, has a very low efficiency. To enhance the probability and intensity of inelastic scattering, the excitation laser wavelength can be selected such that it matches the electronic transition of the molecules of interest, significantly boosting the signal by a factor of 10^2^ to 10^6^ in a process known as *resonance* Raman scattering.^13^-^15^

Even greater enhancement factors can be produced via a different mechanism. Molecules placed in close proximity to a plasmonic material, such as a metal nanostructure, experience both light-molecule and light-metal interactions that affect the Raman scattering cross section. The coupling of these two interactions greatly enhances the inelastic scattering efficiency through the phenomenon we refer to as SERS.^16^-^19^ When the excitation laser is incident upon a metal-dielectric interface, the non-localized conduction electrons of the metallic nanostructures can be stimulated into collective oscillation. If the frequency of the incident excitation matches the intrinsic oscillation frequency of the delocalized electrons, surface plasmon resonance (SPR) will be triggered. For metallic nanostructures, SPR is highly confined by the geometry of the nano-structure, leading to localized SPR (LSPR).^20^-^23^ In fact, LSPR has been recognized as the predominant mechanism contributing to SERS enhancement. LSPR generates nanoscopic areas with intense electromagnetic field, termed as hotspots, with enhancement factors ranging from 10^4^ to 10^11^.

As an optical spectroscopic technique, label-free (or intrinsic) Raman imaging, without the use of nanoparticles as contrast, has been explored clinically for delineating cancerous tissues from healthy tissues based on differences in the Raman spectral fingerprints. However, as a result of the very limited Raman scattering of tissues and the consequent weak Raman signal, long acquisition time is needed for intrinsic Raman imaging. This compromises the feasibility of intrinsic Raman spectral imaging for most biomedical settings and limits its clinical use. For real-time *in vivo* imaging, the Raman signal intensity needs to be significantly amplified, which can be achieved with the use of exogenous contrast agents that provide more intense spectra than the intrinsic Raman signal.^24^-^29^ These contrast agents typically comprise plasmonic nanoparticle cores, Raman reporter molecules, dielectric shells, and ligands against specific targets, or other functional moieties, as shown in **Fig. 1**. The enhancement of the SERS signal of exogenous contrast agents requires the optimization of several parameters, particularly the type, shape, and size of the metal nanoparticle core, the quantity and optical absorbance of Raman reporter molecules attached to the nanoparticle, the composition and thickness of shell layer, and the selection of excitation laser wavelength. Raman imaging with SERS nanoparticles has shown excellent sensitivity, high specificity, low background, and multiplexing capability.^30^-^37^

Among the several plasmonic substrates capable of generating SERS enhancement, such as gold, silver, and copper nanomaterials, gold nanostructures stand out due to their biological inertness, which promises a greater potential for clinical translation.^38^-^45^ In addition, of the different shapes of nanostructures, such as spheres, rods, cubes, prisms, and pyramids, the star-shaped nanoparticles (i.e., nanostars) (**Fig. 2c**) have been shown to generate greater SERS enhancement when excited with NIR lasers.^46^-^51^ In order to preserve the intense and distinct Raman fingerprint in the physiological environment, an encapsulating matrix, such as silica or polyethylene glycol (PEG) shell,^52^-^55^ is often used in order to stabilize the nanomaterial core and protect the Raman reporter molecules against desorption and degradation. To further increase the SERS intensity, the resonant interactions at the metal-reporter interface can be maximized. To this end, Raman reporter molecules with a strong affinity to the metallic core and an optical absorbance resonant with the excitation laser (**Fig. 2d**) can be selected to yield SERRS nanoparticles with superior Raman scattering intensity.^47^, ^56^ A promising new class of Raman reporter molecules, with a chalcogenopyrylium structure, has been reported in the literature. Recent studies comparing the Raman signal of different reporter molecules conjugated to gold nanoparticles,^57^, ^58^ showed that chalcogenopyrylium-based dyes yielded more intense Raman signal than the commercially available non-resonant dyes BPE and AZPY (**Fig. 2e** and **Fig. 2f**). The markedly greater signal enhancement was achieved due to the stronger conjugation between gold nanoparticles and chalcogenopyrylium dyes facilitated by the presence of thiols and attractive electrostatic forces, as well as the dye absorbance tuned specifically to match the laser excitation wavelength. Because of these considerations, to date, one of the best performing SERRS contrast agents with exquisitely bright signal has been constructed from a 60- to 70-nm gold nanostar core, functionalized with specific Raman reporter having a resonant frequency at about 785 nm, and encapsulated within a silica shell, resulting in nanoparticles of about 110 nm diameter (**Fig. 2g**).^47^, ^59^

As with most nanoparticles with a similar overall size, SERRS nanoparticles are able to accumulate preferentially at tumor sites because of the enhanced permeability and retention (EPR) effect. However, SERRS nanoparticles may also be functionalized with antibodies or other ligands, including peptides and aptamers, to achieve active targeting of molecules of interest, such as cell receptors implicated in cancer (**Fig. 1**). For example, SERRS nanoparticles have been conjugated with folic acid, or cyclic Arg-Gly-Asp (cRGD) peptide to specifically target folate-receptor-expressing ovarian tumor and integrin-expressing brain tumor, respectively.^60^-^62^ They have also been conjugated with tissue-factor targeting antibodies to detect pulmonary micrometastases to the lung.^63^ As nanoparticles may not be readily excreted from the body, it is highly desirable to formulate SERRS contrast agents with negligible toxicity - a necessary prerequisite to facilitate their regulatory approval and clinical translation. As such, the physicochemical properties (e.g., size, charge, surface functionalities, etc.) and biological behaviors (e.g., toxicity, stability, immunocompatibility, biodistribution, clearance mechanisms, etc.) of SERRS nanoparticles need to be precisely tuned to meet the requirements set by the regulatory bodies.

## Instrumentation

The successful detection of SERS contrast agents *in vivo* is reliant not only upon their brightness and targeting efficiency, but also on the capabilities of the Raman imaging instrumentation used. To ensure efficient imaging and successful tumor detection, several factors related to Raman instrumentation and the specific intended final application must be considered. These factors include laser wavelength and power, objective, grating, choice of detector (typically a charge-coupled device (CCD)), acquisition speed, and depth of measurement.

When selecting the optimum laser wavelength for Raman imaging, it is important to minimize interference from tissue fluorescence and absorption of incident light. This can be achieved by selecting a laser wavelength within the NIR optical window which allows greater penetration of light into tissues, typically a diode laser at 785 nm.^64^, ^65^ The intensity of the incident light must also be considered in order to avoid burning the sample; this can be circumvented in part by utilizing a larger spot size as this reduces the overall power density, albeit at a lower spatial resolution.^66^

The choice of objective is also extremely important as the laser spot size and consequential power density is also influenced by the objective optics; it is important to select an objective which allows adequate spatial coverage and resolution while avoiding sample degradation and burning.^67^ Lower magnification/low numerical aperture (NA) objectives are capable of imaging deeper into a sample (z focal-plane) due to the longer objective working distance, but are also associated with decreased spatial resolution.^67^ Higher magnification/high NA objectives provide higher spatial resolution; however, their shorter working distance makes them less suitable for working with animal subjects, from both an ease of use and depth of imaging perspective, and are therefore typically better suited to thin samples such as *ex vivo* tissue sections.

Diffraction gratings play an essential role in dispersing the light onto the detector and are defined by the number of grooves per mm on the surface (typically 150 - 4000 per mm).^67^ A higher groove frequency is associated with higher spectral resolution at the expense of reduced intensity and spectral range detected by the CCD.^67^ A CCD is composed of an array of detector elements that are electrically biased so that they generate and store electric charge when exposed to light; as the scattered light is diffracted by the grating based on its frequency, each CCD element receives light from a specific frequency band.^68^ The amount of charge associated with each detector element pixel directly relates to the number of photons illuminating the pixel, which is then “read out” by changing the electrical bias of an adjacent capacitor and eventually assigning a numerical value. The Raman spectrum (example shown in **Fig. 2b**) is a plot of the numerical value (counts) associated with each pixel on the CCD and it is important that a CCD demonstrates high quantum efficiency (conversion efficiency) and high signal-to-noise ratios (SNR).^65^ Achieving a high SNR is particularly important when performing *in vivo* Raman imaging. In general, SNR is influenced by three main sources: dark-noise, read-out noise, and shot-noise. All three of these sources are important factors to consider and steps must be taken to minimize the contribution from each source of noise. For example, if one wishes to increase the mapping speed through reduction in acquisition time (increased read-out speed) read-out noise will be the predominant factor which will in turn lower overall SNR.^69^ For a more detailed discussion on each of these three sources as well as means to improve SNR, we direct the reader to the following articles where they are discussed at greater length.^65^, ^67^, ^69^

When selecting Raman instrumentation for *in vivo* imaging, it is extremely important to select a system that supports the intended application most efficiently. Traditionally, pre-clinical imaging with SERRS nanoparticles is carried out using advanced confocal Raman microscope systems in which the animal, typically a mouse, is placed on a mapping stage which runs perpendicular to the microscope objective.^47^, ^70^ Such instruments are typically equipped with multiple objectives thus allowing the user to select the most appropriate magnification and resolution. Raman signal is acquired through the objective, typically using a point-by-point scanning approach. As such, the use of high NA objectives which limit the field of view (FoV) severely influences the overall time taken to map an area of interest - a critical factor when working with living subjects under anesthesia (**Fig. 1**, lower right). A different approach to expedite imaging and provide fast Raman mapping is the use of global imaging. In this technique, a large area is illuminated and all spatial points of the image are collected simultaneously on a 2D detector, typically a CCD, but only at a single detection wavelength.^71^, ^72^ In doing so however, the molecularly specific Raman 'fingerprint” spectrum is lost giving rise to a reduction in selectivity and limiting the potential for multiplexed imaging applications.

**Figure 3:**
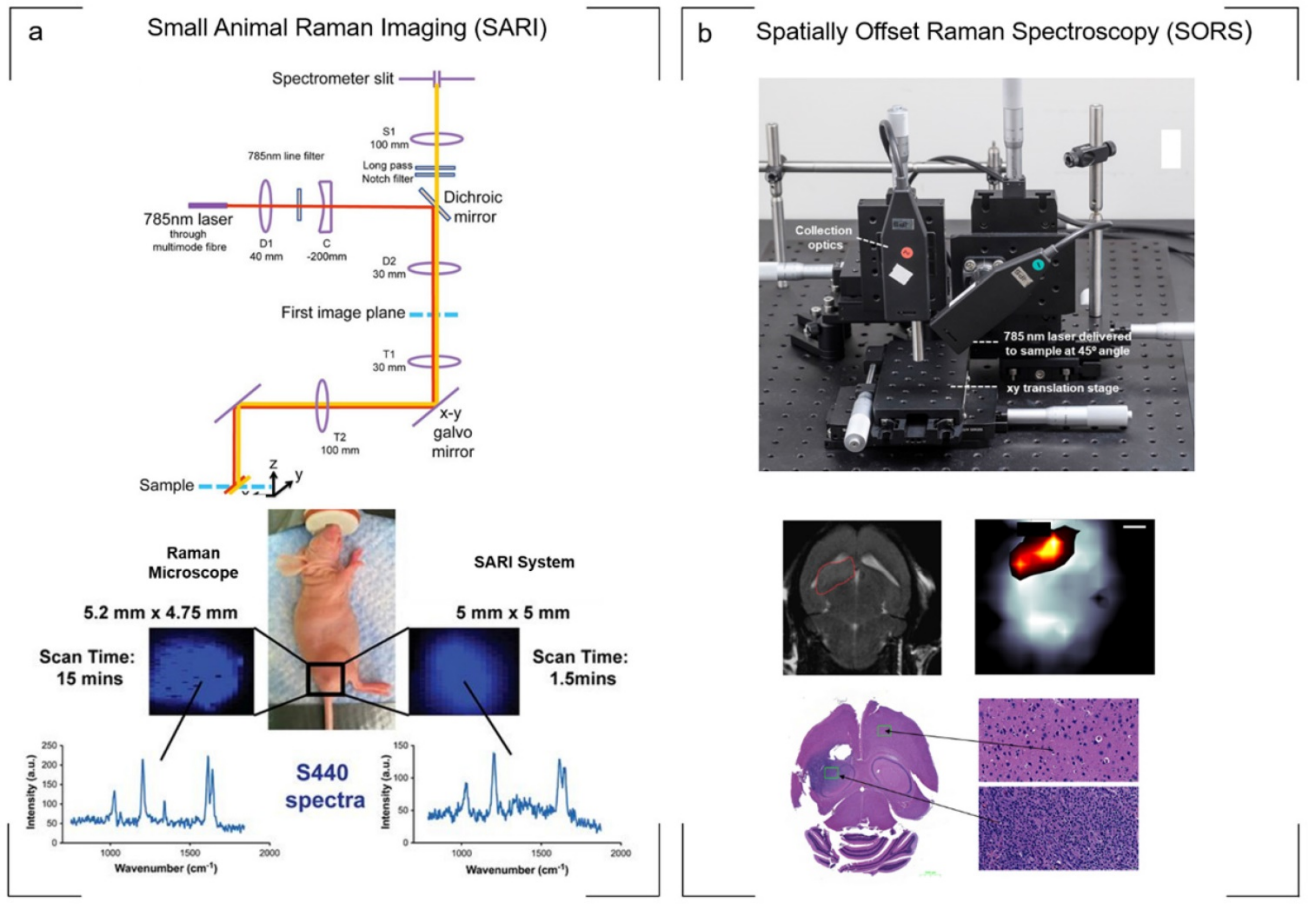
** Raman instrumentation.** (a) Illumination pathway and collection pathway of the Small Animal Raman Imaging (SARI) instrument. The 785 nm laser excitation path is indicated by the red line and Raman scattered light by the yellow line. The SARI provides a 10-fold improvement in scan time compared with a traditional Raman microscope operating in the high-speed acquisition mode with matched spectral and spatial resolution. (b) *In vivo* SORS set up. A 785 nm laser was delivered at a 45˚ angle with regards to the collection optics. A translational *xyz* stage was used to move the laser away from the point of collection in order to apply the SORS technique. Detection of GBM *in vivo* through the intact skull is achieved using SESORS imaging as confirmed by MRI and *ex vivo* histology. (a) Reproduced with permission from ref. 73. Copyright 2013 United States National Academy of Sciences. (b) Reproduced with permission from ref. 89. Copyright 2019 Ivyspring International.

A potential compromise was proposed by Bohndiek *et al*., who applied to the development of a small animal Raman imaging (SARI) unit for the imaging of larger areas spanning the size of the animal (**Fig. 3a**). Using line-scanning in combination with a raster scanning approach, the authors reported the ability to perform fast, spectroscopic imaging over a wide field of view (> 6 cm^2^) at an order of magnitude faster than existing microscopy systems, all while maintaining the selectivity, multiplexing capabilities, and spectral and spatial resolution associated with point-by-point Raman imaging. Specifically, in comparison to a commercially available Raman microscope system utilizing a “fast scanning mode”, the SARI unit demonstrated a 10-fold improvement in scan time (1.5 min vs. 15 min) and a 240-fold improvement in speed of acquisition when compared to the traditional raster scan mode (1.5 min vs. 360 min).^73^

Handheld wide-field Raman scanners have also been reported and have been shown to be capable of capturing the fingerprint region of a 25 mm^2^ field of view, with a spatial resolution <100 µm and an average spectral resolution of 95 cm^-1^ in as little as 90 seconds.^74^ These systems however are only suited to surface-based lesions and thus fail to probe through larger, more clinically relevant depths.

The need for Raman-based detection and imaging of tumors on the internal epithelial body cavities has led to the development of portable fiber optic Raman endoscopic probes. A clinically translatable Raman endoscope has been developed for the detection of topically applied SERS nanoparticles,^75^-^79^ and also more recently, for the detection of pre-malignant lesions of the gastrointestinal (GI) tract.^80^ Using a commercially available Raman probe capable of being inserted into the accessory channel of a clinical white-light endoscope to enable simultaneous dual-modal white-light/Raman imaging, Harmsen and colleagues demonstrated that SERRS contrast agents are capable of detecting pre-malignant lesions as small as 0.5 mm along the GI tract.^80^ The scanning Raman endoscope included a rotating mirror to enable circumferential distribution of the laser along the intestinal epithelium. A pre-clinical dual mode (Raman and fluorescence) endoscope for the detection of nanoparticles with fluorescence and surface-enhanced Raman scattering contrast (F-SERS dots) has also been reported.^81^, ^82^ The detection of SERS contrast agents *in vivo* using handheld fiber optic probes has been reported for the imaging of immune response^83^ as well as image guided resection of glioblastoma multiforme (GBM).^84^ The handheld probe enabled real-time scanning and identified microscopic foci of cancer in the resection bed that would have otherwise been missed.^84^

Although highly useful for detecting the microscopic spread of cancer inside bodily cavities, endoscopes based on fiber optic Raman probes suffer the same weaknesses as traditional Raman microscope systems: they cannot detect the signals though large thicknesses of tissue. This is due to light scattering from the tissue, which diffuses the light in all directions at random and impedes its detection by the objective. Taking into account the principle of photon migration in turbid media, “spatially offset Raman spectroscopy” (SORS) permits the acquisition of signal through significantly greater tissue thicknesses in comparison to conventional Raman optical configurations while also suppressing autofluorescence contributions from tissue.^85^, ^86^ Termed “surface-enhanced spatially offset Raman spectroscopy” (SESORS), SORS has also been used to detect SERS nanoparticles through 50 mm of tissue in phantom studies^87^ using a transmission-SORS approach in which the inelastically Stokes scattered light was collected on the opposite side of the sample (180º to the incident laser light). Other SORS configurations include ring collection SORS as well as ring illumination SORS (termed inverse SORS). In this instance, an axicon lens is used to deliver the laser in a ring configuration onto the sample surface and the scattered light is collected from the center of the illumination ring.^86^ In comparison to a focused beam, the use of a defocused beam or ring illumination permits higher laser powers to be delivered to the sample surface, supporting more favorable permissible exposure limits.^86^, ^88^, ^89^ The use of SESORS for the non-invasive detection of cancer *in vivo*, specifically GBM, was recently demonstrated using a custom built SORS system. In this instance, two fiber optic probes were spatially offset from each other with one probe delivering a diffuse beam to the intact skull while a second probe detected the scattered Raman photons from the SERRS nanoparticles which had accumulated within the tumor through the skull^89^ (**Fig. 3b**). Moving forward, SESORS offers the potential to detect tumors at much deeper levels than what can currently be achieved by offering the ability to detect specific molecular information at depths far superior to standard Raman imaging techniques.

## Non-Targeted SERS Nanoparticles for *In Vivo* Imaging of Premalignant Lesions

One of the ongoing goals in the development of imaging techniques for early cancer detection is to precisely delineate the compositional differences between tissues, distinguishing malignant and premalignant lesions from normal tissues.^90^-^93^ With its unparalleled signal specificity and extremely high sensitivity, *in vivo* Raman imaging with SERS nanoparticles can be potentially exploited for earlier cancer detection than currently possible with other imaging techniques. This would significantly aid curative intervention, improving the prognosis and therefore, the quality of life of cancer patients. To that end, studies have demonstrated that SERS imaging is capable of locating neoplastic lesions at the premalignant stage prior to turning cancerous.^47^, ^80^

**Figure 4:**
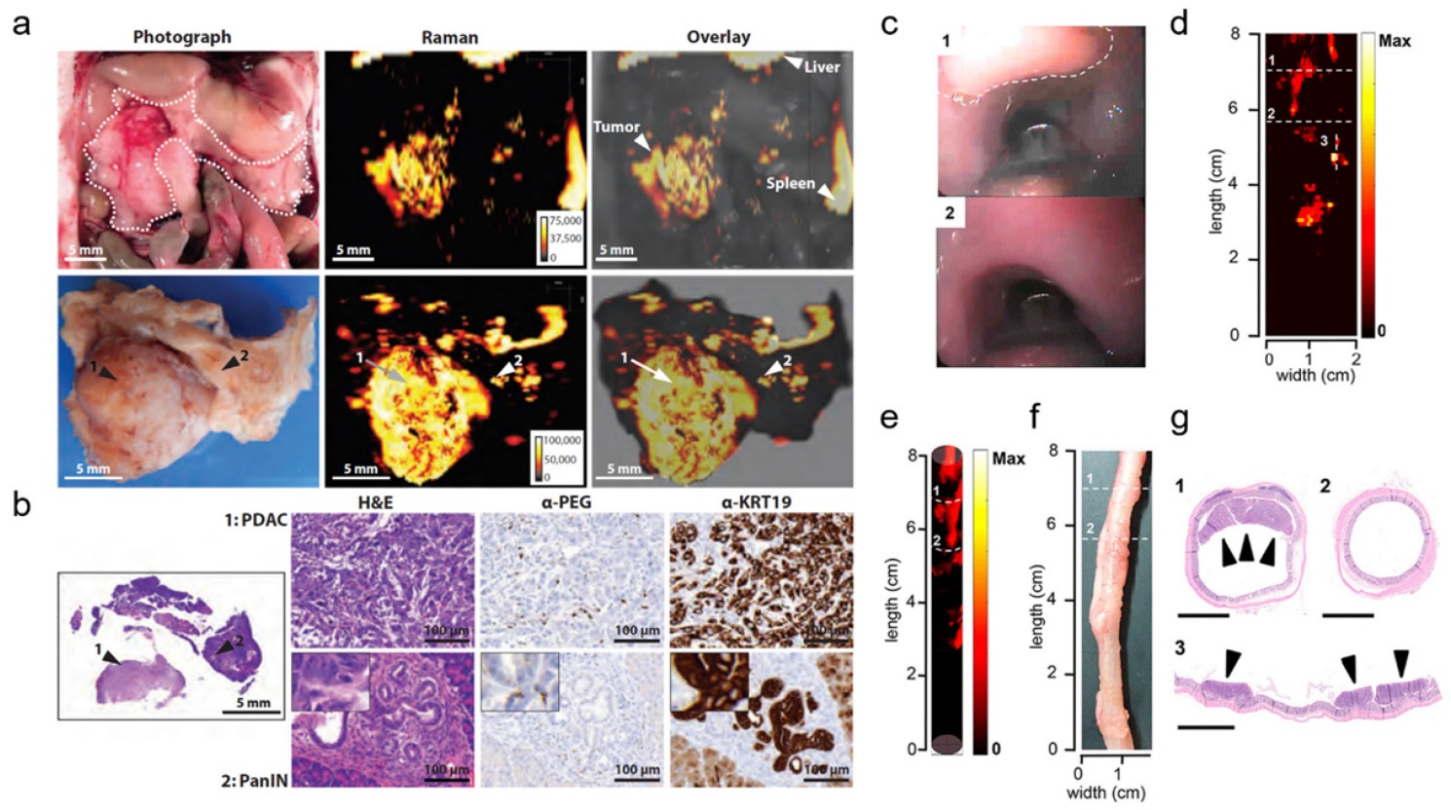
** Untargeted SERRS Nanoparticles for Detection of Premalignant Lesions.** (a-b) SERRS imaging of premalignant lesions in a KPC pancreatic cancer mouse model. (a) Photograph and the corresponding Raman images of the upper abdomen of a mouse with a pancreatic ductal adenocarcinoma (PDAC) in pancreas (top panels, outlined with a white dotted line) and excised pancreas (bottom panels) (b) H&E staining of the pancreas indicating PDAC and pancreatic intraepithelial neoplasia (PanIN) (arrows 1 and 2, respectively). Lesions in regions 1 and 2 were confirmed with histology and keratin 19 (KRT19) staining (c-g) SERRS imaging of premalignant lesions in an Apc gastrointestinal cancer mouse model. (c) Endoscopic images of polyp and normal tissue (labeled as “1” dashed line region and “2”, respectively) in the colon of a rat. (d) 2D Raman map of the SERRS signal intensity along the colon of the rat. (e) 3D projection of the 2D Raman map of the same colon of the rat. (f) White-light image of the evaluated colon *ex vivo*. (g) Histopathologic examination of the colon confirmed the correlation between positive SERRS signal and the presence of adenomatous polyps (labeled as “1” and “3”) as well as negative SERRS signal and the absence of lesions (labeled as “2”). Scale bars represent 2.5 mm. (a-b) Adapted with permission from ref. 47. Copyright 2015 American Association for the Advancement of Science. (c-g) Adapted with permission from ref. 80. Copyright 2019 American Chemical Society.

Several applications of Raman spectroscopic imaging using non-targeted SERRS nanoprobes for delineating premalignant lesions *in vivo* have been reported by the Kircher group.^47^ In one of these studies, SERRS nanostars comprising a 75-nm star-shaped gold core, a NIR-resonant Raman reporter, and a PEGylated silica shell were employed. The SERRS nanostars demonstrated femtomolar sensitivity in phantoms and were reported to accurately detect the full sequence of cancer progression, particularly the premalignant stage, in murine models of pancreatic, prostate, and gastrointestinal cancer. For example, in a KPC pancreatic cancer mouse model, the presence of pancreatic intraepithelial neoplasia (PanIN) - a precursor of invasive pancreatic ductal adenocarcinoma (PDAC) - could be detected in small submillimeter foci in the body and tail of pancreas based on the SERRS signal (**Fig. 4a**), as verified by histology (**Fig. 4b**). Histological evaluation of the bulk tumor revealed the accumulation of SERRS nanostars in both the tumor stroma and within epithelial tumor cells. Similarly, the existence of premalignant high-grade prostatic intraepithelial neoplasia in a Hi-Myc prostate mouse model could be determined in the prostate, based on signal from the SERRS nanostars. The successful delineation of precancerous tissues in both murine carcinogenesis models was made possible because of the selective passive accumulation of the nanoparticles within the submillimeter premalignant lesions, which in turn, was attributed to the macropinocytosis of the nanostars by the neoplastic cells.

In one of the more recent works of the Kircher group, non-targeted SERRS nanoprobes were utilized in conjunction with a commercial Raman imaging system, a clinically validated Raman endoscope, and a white-light endoscope to detect and delineate premalignant dysplastic gastrointestinal tract lesions in rat models of esophageal, gastric, and colorectal cancer (**Fig. 4c-g**).^80^ Similar SERRS nanostars were used, with a 60-nm gold core, Raman reporter, and a 15-nm silica shell passivated with the hydrophilic polyethylene glycol (PEG) layer, and featured a distinct spectral or diagnostic peak at 950 cm^-1^. The developed imaging system relied on white light to visualize relevant macroscopic anatomical context (**Fig. 4c**) and SERRS signal to locate colorectal polyps (**Fig. 4d**). The correlation between the SERRS signal and the colorectal polyps was demonstrated through a three-dimensional cylindrical projection (**Fig. 4e**) of the two-dimensional Raman map (**Fig. 4d**) and the histopathologic examination of the SERRS-positive lesions (**Fig. 4f**, **g**; 1,3). Areas with no SERRS signal (**Fig. 4d**, **e**; 2) were found to correspond to normal colorectal tissues (**Fig. 4f**, **g**; 2). SERRS nanoparticles were reported to accumulate uniformly in the premalignant gastrointestinal tumors of the esophagus, stomach, and intestines, and provided sufficient sensitivity, which made the detection of these precursor lesions possible. This approach could significantly facilitate targeted biopsies and improve therapeutic intervention.

## Molecularly Targeted Tumor Imaging

Accumulation of SERS nanoparticles at the target site leads to a localized increase of the Raman signal intensity, generating contrast against surrounding tissues, which gives SERS its imaging capability. Thus, the pharmacokinetic profile of SERS nanoparticles is a crucial factor for their utility as contrast agents. The selective accumulation of a contrast agent into tumors depends on the agent's ability to extravasate into the tumor region and also on its molecular targeting specificity, allowing the detection and retention upon specific cell types or sub-types.

One approach, as we discussed above, is to rely solely on the nanoparticle biodistribution as a delivery mechanism, without targeting moieties. This technique, known as passive targeting, exploits the physiological properties of tumors, such as the poorly constructed networks of “leaky” neovasculature and the decreased lymphatic clearance, both of which contribute to EPR effect. However, reliance on passive nanoparticle accumulation is complicated by heterogeneity in vascularization, both inter- and intra-tumoral,^94^ and the inability to accurately direct or advance retention of the contrast agents within the tumor microenvironment.^95^ It is generally established that with such techniques, only a small fraction of the injected nanoparticles reach the tumor, as these nanomaterials are recognized as foreign and sequestered by the body's defense mechanisms.^96^ Upon introduction of nanoparticles to the biological environment, non-specific biomolecules may adsorb and a protein corona (PC) may form on the nanoparticle surface. The composition of the PC, dictated by the physicochemical properties of the nanoprobes, in turn shapes the circulation lifetime of the nanoparticles before eventual uptake into the mononuclear phagocytotic system (MPS).^97^ PEG has long been used to increase the nanoparticle hydrophilicity, with a high density, brush-like structure reducing the rate of protein adsorption and therefore opsonization.^98^, ^99^ To further increase nanoparticle bioavailability, the combination of a silica shell and PEG coating has also been found to improve upon the deterrence of PC formation and subsequent macrophage clearance.^100^ Another strategy used to pre-empt this mechanism is by using fragments of cell membrane as a stealth coating for nanoparticles, allowing them longer circulation times.^101^

A different approach is to improve the retention of nanoparticles at the tumor through the functionalization of their surface with targeting ligands that bind a molecule of interest, often a cell receptor. This approach, called “active targeting”, aims to not only increase nanoparticle affinity to the tumor but also confer specific imaging of the chosen target. Various targeting molecules have been shown to be effective, such as aptamers, antibodies or antibody fragments, and small molecules. Ideally tethered to the distal end of the PEGylated chain surface,^102^ receptor-mediated targeting increases nanoparticle uptake by increasing affinity and specificity. A meta-analysis study conducted by Wilhelm et al. found that employing active targeting increases both the delivery and retention efficiency of nanoparticles, compared to passive targeting, within the intratumoral environment.^96^

Specifically, active targeting can be used to improve the tissue imaging capability of Raman imaging with SERS contrast agents. To this end, it is instrumental to modify the surface of nanoparticles with targeting moieties capable of tethering the SERS contrast agents to the tumor microenvironment with high affinity and specificity. This approach aims to enhance the overall concentration of SERS nanoprobe at the target site thereby enhancing the intensity of SERS signal. Such functionalized nanoprobes have been widely explored to target the biomarker receptors, which are overexpressed on the surface of several cancer cells, such as folate receptor, epidermal growth factor receptor (EGFR), transferrin receptor, asialoglycoprotein, low-density lipoprotein receptor, etc.^103^-^107^ Antibodies and their fragments have long been the traditionally used active targeting moiety. Highly specific recognition of the increased expression of EGFR within head-and-neck squamous cell carcinoma (Tu686) in xenograft mouse models was demonstrated by Quian et al. using SERS gold nanoprobe conjugated with tumor targeting ligand such as single-chain variable fragment (ScFv)-EGFR antibody.^106^ The human epidermal growth factor receptor 2 (HER2) is an important receptor overexpressed in most solid breast tumors, which plays an important role in cell proliferation.^108^ In another study, Samanta et al. used SERS to demonstrate the unique targeting capability of Au nanoparticles functionalized with lipoic acid-containing NIR-active tricarbocyanine (CyNAMLA) and scFv anti-HER2 antibodies in HER2-positive SKBR-3 xenograft models, in comparison to the HER2-negative MDA-MB-231 models.^107^

Aptamers, either based on peptides or oligonucleotides, are a preferred targeting ligand by many researchers. Having good efficacy and safety in humans, aptamers are generally of low cost and require few simple steps to adhere to the nanoparticle surface while maintaining their highly selective outward binding capabilities. Mucin1 (MUC1) is a common target for aptamers as it is overexpressed in the vast majority of human breast carcinomas, including in the early stages of triple negative breast cancer, and plays a role in the progression and metastatic potential of a tumor lesion.^109^ To confirm the homing ability of targeted SERS nanoparticles, Pal et al. studied the competitive uptake of solely PEGylated and DNA aptamer functionalized nanoparticles.^110^ Nude mice were simultaneously implanted with two xenograft tumor models with one lesion overexpressing MUC1 and a MUC1-negative control. The distinctive SERS signal of the Raman reporter, bound to the gold surface of the DNA aptamer functionalized nanoprobe, was detected more strongly within the MUC1 positive lesion, confirming selective uptake. The integrin αvβ3 is highly expressed on the surface of neovasculature and is implicated in metastatic invasion commonly found in glioblastomas and melanoma. Arg-Gly-Asp (RGD) peptides bind with high affinity to this extracellular matrix receptor.^111^ Nicolson et al. published the first *in vivo* SESORS study in 2019, functionalizing the SERRS nanoprobe surface with cyclic-RGDyK peptide to non-invasively image induced glioblastoma multiforme tumors within the RCAS-PDGF/N-tva transgenic mouse model.^89^ Although novel analogues are improving their pharmacological profile, native peptides have intrinsically weak chemical and physical stability, often translating to a poor circulation time and prompt elimination by the MPS.^112^

**Figure 5:**
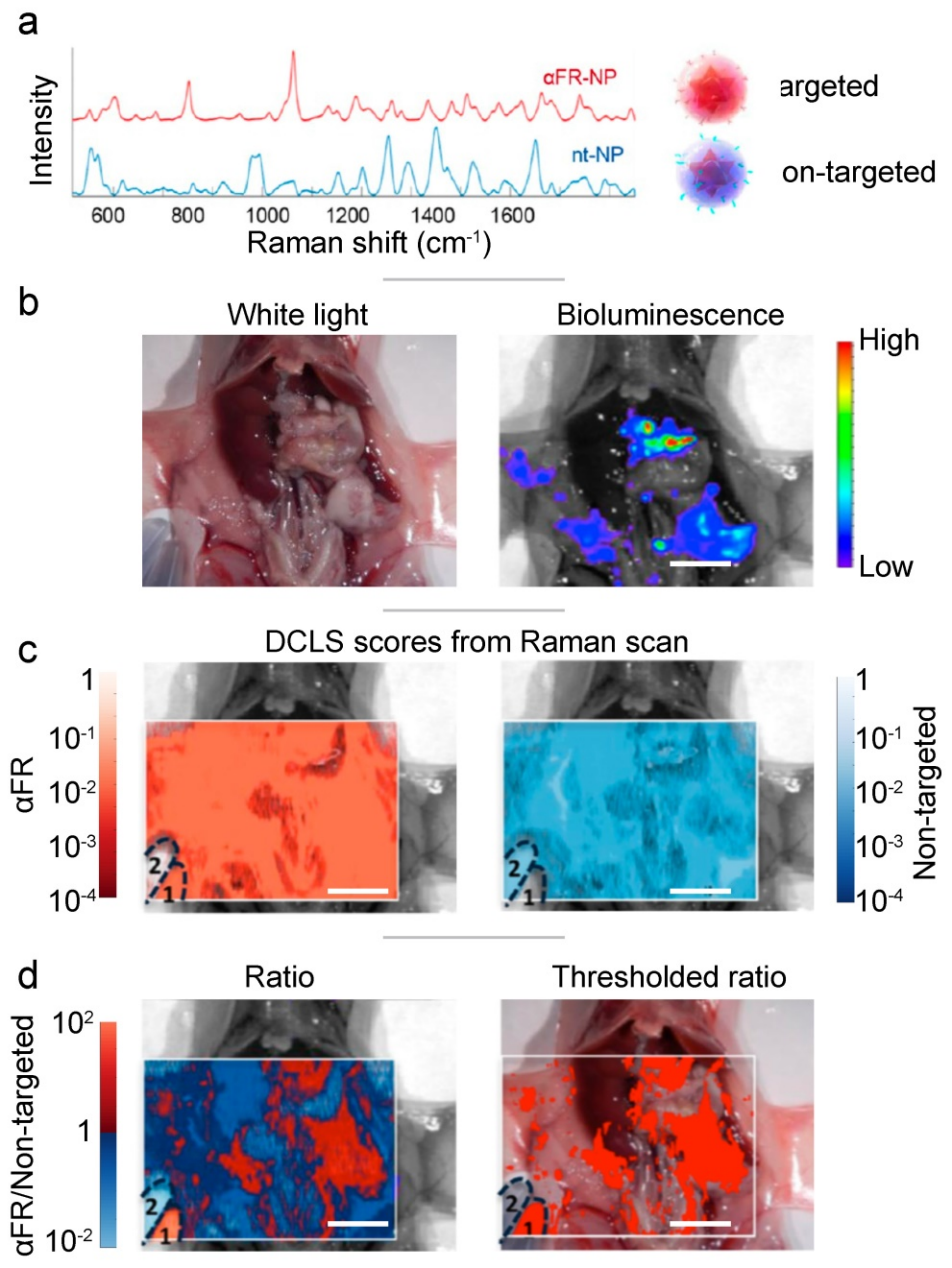
** Topically applied SERRS ratiometry targeting the folate receptor.** (a) Two distinct SERRS nanoparticles were synthesized: red - targeted against the folate receptor, blue - non targeted. (b) White light and bioluminescence imaging make the precise identification of the diffuse tumor difficult. (c) The decoupled signal from each of the two SERRS nanoparticles, does not offer any indication of the tumor site. (d) Ratiometry of the targeted probe over the untargeted, reveals the extend of the main tumor and multiple microtumors. Adapted with permission from ref. 60. Copyright 2017 American Chemical Society.

In addition to the clinical markers mentioned above (i.e., EGFR, HER2), the folate receptor is a popular choice for cancer imaging. During the disruptive, fast-laying of neovasculature, folate receptors are highly expressed on the cell surface in a number of cancers including breast, lung and ovarian.^113^ Feng et al. functionalized SERS nanoprobes (gold nanobipyramids) with PEG-folic acid to target the folate receptors present on MCF-7 breast cancer cells, injected subcutaneously in Balb/c (nu/nu) nude mice.^114^ The *in vivo* study confirmed the specific accumulation of the targeted nanoprobes within the tumor. For the detection of metastatic spread of ovarian tumors within the peritoneal cavity, Oseledchyk et al. challenged athymic mice with human ovarian adenocarcinoma cell line SKOV-3 (**Fig. 5**).^60^ Both non-targeting and anti-folate receptor targeting SERRS nanoprobes were topically applied, and a SERRS ratiometric algorithm revealed the areas of overexpressed folate receptor. Alongside histological and biodistribution analysis, this SERRS imaging technique confirmed the increased retention property of the nanoprobe granted by the folate receptor targeting functionalization. As the nanoprobes were applied topically (directly within the peritoneal cavity), they did not need to circulate through the blood stream, avoiding sequestration by the immune system and also eliminating potential toxicity to off-target tissues.

Active targeting provides an increased internalization and retention of the nanoprobes within the tumor microenvironment and facilitates the ability to conduct non-simultaneous multimodal imaging. Additionally, active targeting is essential in selectively highlighting areas that express specific molecules of interest within the tumor microenvironment, allowing the imaging of multiple molecular targets by using a library of highly specific Raman reporters and their corresponding targeting ligands.

## Multimodal SERRS Nanoparticles for *In Vivo* Delineation of Localized Tumors

While Raman imaging with SERS nanoparticles is considered a powerful emerging medical imaging technique, it suffers from several inherent technological limitations such as poor tissue penetration, small field of view, and long image acquisition times compared to other imaging techniques. These shortcomings can be remedied by adding additional modes of contrast to the SERS nanoparticles, to allow for multimodal imaging.

Medical imaging with complementary modalities is now common clinical practice, as it allows acquisition of images with integrative information. The most common clinical example is the use of PET-CT, which combines the molecular specificity of immuno-PET with the high-resolution physiological imaging of CT.^115^ New multimodal approaches could allow for non-invasive macroscopic imaging for surgical planning as well as for microscopic intra-operative imaging for defining the tumor margins with a single injection of contrast. Combinations of imaging modalities can help avoid possible ambiguities stemming from a single method, as well as overcome the limitations in contrast and resolution of the independent imaging techniques.

**Figure 6:**
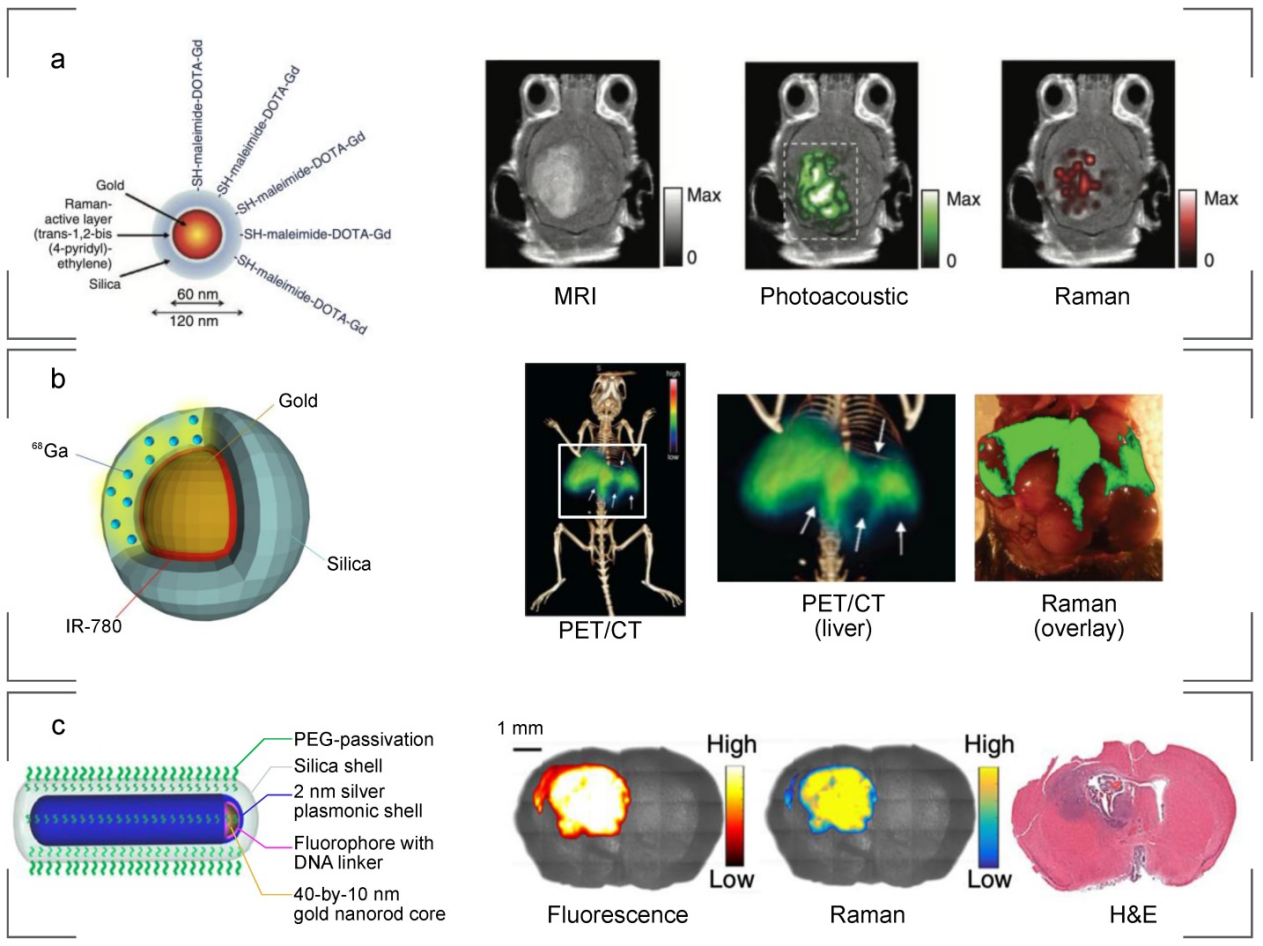
** SERS nanoparticles developed for multimodal imaging.** (a) A tri-modality MRI/Photoacoustic/Raman nanoparticle. The gold core provides photoacoustic contrast while Gadolinium, chelated on the surface, provides MRI contrast. (b) A PET/Raman nanoparticle. Gallium-68 embedded within the silica shell emits positrons allowing pre-operative whole-mouse imaging. The contrast comes from healthy liver tissue, with hypo-intense tumors. (c) A fluorescence/Raman nanoparticle based on a DNA linker for the fluorophore allows quick intraoperative tumor location with fluorescence, and highly precise margin definition with Raman imaging. (a) Adapted with permission from ref. 116. Copyright 2012 Nature Publishing Group. (b) Adapted with permission from ref. 133. Copyright 2017 Wiley-VCH. (c) Adapted with permission from ref. 110.

In multimodal SERS imaging, a precisely functionalized nanoparticle acts as a contrast agent for Raman imaging via SERS or SERRS, as well as for various other medical imaging modalities such as photoacoustic imaging (PAI), MRI, fluorescence imaging, PET, CT, etc. (**Fig. 6**). During multimodal tissue imaging, a single dose of administered multimodal imaging contrast facilitates the surgeon to precisely correlate tumor margin in pre- and intraoperative images, which can help to more precisely identify the tumor extent, while also potentially limiting the toxicity associated with administration of multiple contrast agents.

In order to develop an efficient multimodal SERS nanoprobe, there are several key design parameters that need to be evaluated, such as size, shape, composition, surface functionalization, etc. The multilayered structure of a SERS nanoparticle, which usually comprises of a plasmonic metal core, a layer of a Raman reporter, and a protective coating layer, e.g. PEG or silica, which can then be functionalized with targeting or other moieties, can readily integrate additional modalities into the structure with minor or no modifications. For example, due to its high extinction coefficient for NIR light, the gold core of a nanoparticle provides high signal intensity for PAI, as was demonstrated in several multimodal imaging reports.^116^-^119^ CT is another modality enabled by the presence of a gold core, as gold is known to provide a higher X-ray contrast than the traditionally administered iodine.^120^ SERS nanoparticles based on gold nanospheres,^121^ nanorods,^117^ Janus nanostars^122^ and even gold-iron alloy^123^ were shown to be successfully used in both Raman and CT imaging. To integrate the MRI modality, it becomes necessary to add a magnetic component to the nanoparticle to provide MR contrast. This can be achieved in various ways, e.g. adding paramagnetic iron oxide in the core of a SERS nanoparticle,^122^, ^123^ adding iron ions in the coating layer;^124^ forming a composite of SERS nanoparticle and superparamagnetic iron oxide nanoparticles^125^ or, more commonly, integrating gadolinium as an MRI contrast agent. The latter is most often attached to the nanoparticle surface through chelating ligands,^126^-^128^ however there is also report of using gadolinium from an attached upconversion nanoparticle as an MRI contrast source.^129^ Kircher et al. demonstrated the possibility of integrating SERS with PAI and MRI for imaging brain tumors using a triple-modality nanoparticle comprised of silica-coated gold nanoparticle functionalized with Raman active molecule and gadolinium (Gd) chelate (**Fig. 6a**).^116^ SERS-PET dual imaging has been reported for both chelator-based^75^ and chelator-free^130^ approaches of nanoparticle design (**Fig. 6b**).

An interesting approach is to use Raman imaging with fluorescence, as these are both optical imaging techniques, but each offers distinct advantages. However, when a fluorescent dye is in close proximity to the gold surface of a SERS nanoparticle, its fluorescence is quenched. Still, nanoparticles with dual contrast, combining SERS and fluorescence, have been developed. First and foremost, one can control the distance between a dye and gold surface using an insulating layer^131^ or a DNA strand to enable dual imaging mode (**Fig. 6c**).^110^ Alternatively, fluorescence quenching can be reduced by decreasing the size of the gold core,^132^ or by using upconversion luminescent-SERS nanoparticles.^117^, ^129^

As contrast for additional imaging modalities can be added independently to the various parts of the SERS nanoparticles, there are now triple-[116, 123] and quadruple-modality^117^, ^122^ nanoparticles, demonstrating that it is feasible to integrate all aforementioned modalities into one single complex nanoconstruct - a universal contrast agent, which will most likely be developed in the near future.

## Advanced Mouse Models

Currently, SERS imaging is confined to the preclinical setting, as no SERS-based contrast agents are approved for use in patients. In oncology, preclinical SERS imaging is performed using murine models: grafted with human tumors (xenograft) or with syngeneic tumors (allograft), or genetically engineered mouse models (GEMMs) that recapitulate the human pathology.^134^ To date, most studies utilize xenograft (ectopic or orthotopic) models that allow for rapid imaging of cancer and the evaluation of SERS contrast agents to target to the region of interest. In the typical xenograft assay, 0.5-1.0 million cells from culture derived from human tumors are injected subcutaneously on the flank of the animal, subsequently forming palpable tumor nodules.^135^ With this in mind, it can be said that xenograft studies are not a true 'mouse model', instead representing a human-in-mouse system in which fully established cancer cells are grown in immunodeficient mice with the support of murine stroma and vascularture.^134^-^137^ Using a different approach, cells from a lysed solid tumor (or from culture) can be injected intravenously, intraventricularly, or orthotopically, to mimic the process of metastasis.^135^ While this approach has notable flaws, its ease of use and low cost have enabled it to be used extensively in the last decade for the testing and validation of SERS contrast agents. However, recent advances in genetic engineering have enabled the development of more sophisticated GEMMs to which SERS contrast agents have also been administered. These mice provide immunocompetent models and possess several desirable features including spontaneous tumor growth *in situ* in the appropriate organ, prior knowledge of the initiating molecular mechanisms, and can generate tumors which possess the architectural and cellular complexity associated with disease as found in patients, i.e. with inflammatory cells, vasculature, and other tumor-stromal interactions required for tumor progression.^134^-^137^

Several GEMMs have been employed to test the uptake of SERS contrast agents *in vivo* (Table 1). The most frequently utilized GEMM reported in the literature and used in SERS imaging is the RCAS-PDGF/N-tva transgenic mouse model of GBM.^61^, ^84^, ^89^, ^110^, ^118^ This mouse model system permits the somatic gene transfer of selected oncogenes, such as PDGF, into targeted brain cells engineered to express the tv-a receptor. These transgenic tv-a mice can then be crossed onto various genetic backgrounds to model the effects of genetic aberrations such as loss of tumor suppressor genes on glioma formation and response to therapy.^138^ Importantly, this GEMM exhibits histopathological and imaging hallmarks of human high-grade tumors such as infiltrating tumor, margins, microvascular proliferation and pseudopalisading necrosis.^138^ In these reports, *Nestin*-tv-a/*Ink4a-arf^-^*^/*-*/^/*Pten^fl/f^*^/^ mice were stereotactically injected with DF-1 cells transfected with RCAS-*Pdgfb* and RCAS-*Cre,* which leads to the overexpression of oncogene Pdgfb and loss of tumor suppressor genes *Ink4a-arf* and *Pten*, giving rise to almost complete penetrance of GBM after four weeks.^61^, ^84^, ^89^, ^110^, ^118^

GEMMs enable researchers to better visualize cancer in a way that more closely resembles its initial formation, progression, and consequential metastasis in patients. The APC^Pirc/+^ rat model was used to determine the ability of SERS contrast agents to detect premalignant lesions of colorectal cancer (CRC).^80^ Loss of function of the tumor suppressor gene adenomatous polyposis coli (APC) is known to initiate neoplastic growth and the development of adenomatous polyps, however it is well understood that such lesions do not progress into metastatic adenocarcinomas.^139^, ^140^ The APC^Pirc/+^ rat model was thus chosen to mimic the development of premalignant lesions of CRC and determine the effectiveness of both SERS endoscopy to detect such polyps.^80^ Uptake of SERS contrast agents into the KPC model of pancreatic cancer has also been achieved, in which SERS imaging successfully detected PanIN, a microscopic premalignant lesion of the pancreas that can progress to invasive ductal adenocarcinoma with 100% penetrance.^47^ Andreou *et al*., evaluated the ability of SERS contrast agents to detect liver cancer using two GEMMs; (1) Myc-driven model and (2) the Ink4A/ Arf^-/-^ model^141^. Myc is commonly over expressed in hepatocellular carcinomas (HCC)^142^ while inactivation of Ink4A/Arf^-/-^ can lead to the development of histiocytic sarcomas, soft tissue sarcomas, and lymphomas.^141^ The authors demonstrated that SERS contrast agents accumulate in healthy liver tissues but not in liver tumors giving rise to high contrast, high resolution Raman imaging. Importantly, using the Ink4A/Arf^-/-^ model, through the use of SERS contrast agents, it was possible to image microscopic cancerous lesions within the liver which were otherwise undetectable using white light sources.

## Multiplexed SERS for *In Vivo* Cancer Imaging

One of the most promising features of SERS for cancer imaging is its potential for multiplexed detection of numerous cancer-related molecular markers,^30^, ^34^, ^35^, ^76^ particularly their distribution within the tumor microenvironment. The simultaneous imaging of multiple biomarkers in real time, in living subjects, with sufficient specificity will be beneficial for improving cancer staging, monitoring treatment response, and screening for cancer recurrence. In fact, the need for multiplexed detection is evident from the rising trend in leveraging numerous imaging modalities on the same patient to obtain more clinically relevant information.^115^ So far, this has been achieved in the clinic primarily through the co-registration of images acquired over a lengthy period of time using a multimodal approach. The commonly used medical imaging modalities have limited capacity for multiplexed imaging: the radionuclides used in PET offer high sensitivity, but they provide photons of the same energy which are thus indistinguishable; CT contrast agents do not have sufficient sensitivity of detection to allow molecularly specific imaging; MRI relies primarily on intrinsic contrast, which again makes the detection of rare and microscopic targets practically impossible. In the preclinical setting, while a higher number of targets (i.e., up to five targets) can be detected using fluorescence imaging,^143^-^145^ it is still mostly limited to *in vitro* setting and several issues, such as overlapping broad emission spectra, photobleaching, and autofluorescence, have complicated its *in vivo* implementation.

Compared to other imaging modalities, SERS imaging does not have these limitations, and can in theory provide high degrees of multiplexed detection, on par with specialized *ex vivo* imaging methods like multiplexed histology,^146^-^148^ or mass spectrometry (e.g. MIBI).^149^, ^150^ The high multiplexing capacity of SERS stems primarily from the narrow Raman bands that appear in the fingerprint-like spectra, which are distinct to individual Raman reporter molecules. SERS imaging, in principle, is capable of visualizing a large number of molecular markers within the same tumor in a single scan.

Initial demonstration of the high multiplexing potential of SERS nanoparticles was provided by Zavaleta et al. in a study where the authors injected 10 distinct SERS nanoparticles subcutaneously in a mouse and imaged them.^30^ The next step to fully exploit the potential of multiplexed imaging is to systemically administer targeted SERS nanoparticles, and image the distribution of several markers at the tumor site. This is yet to be reported in the literature. To realize this, Raman reporters with distinct spectral signatures have to be synthesized and introduced into identical nanoparticles functionalized with differentially targeting moieties to generate a cocktail of spectrally unique and molecularly targeted SERS nanotags.

One of the earliest studies that reported the utilization of SERS for *in vivo* multiplexed cancer detection with bioconjugated SERS nanoparticles was able to detect three intrinsic cancer biomarkers, i.e., EGFR, CD44, and TGFβ receptor II, in a breast cancer-bearing murine model, after intratumoral injection.^151^ The SERS contrast agents were constructed from different reporter molecules, namely Cy5, malachite green isothiocyanate, and rhodamine 6G, and conjugated with antibodies to enable targeting of the specific cell surface receptors. To date, no studies have been reported that use more than two distinct and targeted SERS nanoparticles administered intravenously.

Encouragingly, ratiometric Raman-encoded imaging has been developed,^60^ and applied to visualize up to four targets *ex vivo*.^152^ With further molecular designs and refinement, SERS imaging can be effectively translated for multiplexed imaging of more than five biomarkers *in vivo*.

## Towards Clinical Translation

The versatile nature of SERS-based contrast agents makes them a highly promising tool in the field of molecular imaging. Their clinical application will serve to complement the existing medical imaging modalities, filling a void between non-invasive imaging methods and molecularly-specific histology performed after biopsy. However, many challenges remain before these nanoparticles are ready for clinical trials; the most important of which include off-target accumulation (in organs of the reticuloendothelial system), potential long-term toxicity effects, and the lack of validated molecular targets reported in the literature.

It is well accepted that most systemically administered nanoparticles with diameters around 100 nm are removed from circulation and retained by the liver and the spleen, leaving only a very small number to reach the target sites.^95^,^153^ Smaller nanoparticles, with diameters well below 10 nm, are instead cleared renally and would pose fewer long-term toxicity concerns.^154^ However, such nanoparticles demonstrate weak SERS, as plasmonic enhancement is strongly related to the number of free electrons (which is proportional to the cubic power of the diameter). One potential alternative approach is to use clusters of ultrasmall nanoparticles which would ideally disintegrate and be cleared through the renal or biliary pathway.^155^

For common gold-core silica-shell SERS nanoparticles, initial *in vitro* studies, by Dr. Gambhir's group, aimed to investigate the toxicology, biocompatibility, and long-term effects and reported minimal toxicity.^156^ Later *in vivo* toxicity studies by the same group reported that after intravenous injection via the tail vein, SERS nanoparticles with a PEG passivation layer were shown to elicit a mild inflammatory response and an increase in oxidative stress in the liver after 24 hours. However, this subsided over two weeks following administration.^157^ A gradual decline in the concentration of gold within the liver of both male and female animals was also observed over the two-week period. Importantly, by measuring clinical, histological, biochemical, or cardiovascular parameters over two weeks, no evidence of significant toxicity was observed.^152^ As part of the same work, the group tried topical administration of the same nanoparticles per rectum. Animals that underwent per rectal administration were also shown to have no significant bowel uptake, or systemic nanoparticle uptake and toxicity.^157^ Results indicated that the non-targeted nanoparticles remained in the bowel lumen and did not cross the colon wall, and in fact, nanoparticles administered per rectally were eliminated from mouse models through their feces. Topical administration of this type of nanoparticles within the peritoneal cavity was also shown to lead to negligible systemic uptake by the Kircher's group.^60^ Although further investigation into the long-term toxicology and biocompatibility of the SERS nanoparticles is certainly required, initial data suggest that SERS nanoparticles have the potential for clinical translation, particularly when administered topically, instead of through the bloodstream.

Validated molecular targeting is also a crucial step in establishing the clinical utility of SERS contrast agents, especially in the context of multiplexed imaging. As unfunctionalized nanoparticles can be taken up by cancer and immune cells, it is important to establish the targeting efficacy of functionalized SERS contrast agents and identify the extent of non-specific interactions. The most common way of validation is by comparison to immunohistological stains after tumor excision, while *in vivo* targeting validation can be shown by using blocking antibodies. A few examples using both validation methods are found in literature, e.g. for nanoparticles targeted against integrin^61^ and against tissue factor^63^. For *ex vivo* imaging several other molecular targets have been validated.

## Conclusion

Raman imaging with SERS nanoparticles makes a highly promising technology for the imaging of cancer. The method has undergone many advancements in recent years, especially when it comes to *in vivo* imaging for preclinical research. Compared to other medical imaging modalities, this technique offers many advantages, mostly stemming from the fact that the contrast agent is a nanoparticle. Nanoparticles enjoy preferential extravasation and uptake in cancerous and premalignant tumors, allowing SERS nanoparticles to reveal these malignancies. Additionally, nanoparticles provide a platform on which functional molecules can be attached, to allow for specific targeting, or for detection by other imaging modalities. The greatest potential of SERS nanoparticles is that they can be engineered to allow detection of multiple targets in a single scan, with high specificity and high spatial resolution *in vivo*. However, for this imaging modality to fulfill its promise, many more advancements are required, both in the realm of instrumentation, to allow for rapid imaging of large and at depth areas, as well as in optimization of the biological interactions of the nanoparticle constructs, acting favorably towards tumors while eluding the MPS and bypassing healthy tissues. As improvements continue on these two fronts, we should continue on the tracks laid by Dr. Gambhir and Dr. Kircher, and push towards clinical approval and translation of medical imaging with SERS nanoparticles.

## Figures and Tables

**Table 1:** Summary of Genetically Engineered Mouse Models used in SERS *in vivo* imaging

Model	Cancer Type	References
APC^Pirc/+^	Premalignant GI tract lesions	80
RCAS-PDGF/N-tva	Glioblastoma Multiforme	61, 84, 89, 110, 118
4Ink4A/ Arf^-/-^	Sarcoma	47, 141
Myc-HCC	Liver cancer	130, 141
MMTV-PyMT	Breast cancer	47
Hi-Myc	Prostate cancer	47
KPC	Pancreatic cancer	47
DDLS	Dedifferentiated liposarcoma	47
